# Soil Aggregation Shaped the Distribution and Interaction of Bacterial-Fungal Community Based on a 38-Year Fertilization Experiment in China

**DOI:** 10.3389/fmicb.2022.824681

**Published:** 2022-03-22

**Authors:** Jie Chen, Dali Song, Donghai Liu, Jingwen Sun, Xiubin Wang, Wei Zhou, Guoqing Liang

**Affiliations:** ^1^Ministry of Agriculture Key Laboratory of Plant Nutrition and Fertilizer, Institute of Agricultural Resources and Regional Planning, Chinese Academy of Agricultural Sciences, Beijing, China; ^2^Institute of Plant Protection and Soil Fertility, Hubei Academy of Agricultural Sciences, Wuhan, China

**Keywords:** soil aggregate, microbial community, co-occurrence network, microbial interaction, long-term fertilization

## Abstract

Soil aggregates provide different ecological niches for microorganisms, and in turn, the microbial interactions affect soil aggregation process. The response of the microbial community in bulk soil to different fertilization regimes has been well studied; however, the co-occurrence patterns of bacteria and fungi in different aggregates under various fertilization regimes remain unclear. Based on the long-term field experiment, we found that fertilization regimes contributed more to fungal than to bacterial community composition. Long-term fertilization decreased microbial interactions in large macroaggregates (LM), macroaggregates (MA) and silt and clay (SC) fractions, but increased in microaggregates (MI). The application of manure with inorganic fertilizers (NPKM) significantly increased the intensive cooperation between bacteria and fungi in LM and MA. Microbial communities in LM and MA were well separated and showed strong competition against microbes in MI and SC; hence, we concluded that the microbial habitat could be divided into two groups, large fractions (LM and MA) and small fractions (MI and SC). The bacterial genera *Anaerolinea*, *Nocardioides*, *Ohtaekwangia*, *Geoalkalibacter*, *Lysobacter*, *Pedomicrobium*, and *Flavisolibacter* were keystone taxa in inorganic fertilization, and *Roseiflexus*, *Nitrospira*, and *Blastocatella* were keystone taxa in NPKM, which were all sensitive to soil aggregation. In this study, we demonstrated that the NPKM decreased the microbial interactions within and between kingdoms in LM, MA, and SC, but enhanced nutrient availability and microbial interactions in MI, leading to the formation of biofilms and the strengthening of stress tolerance, which finally stimulated the formation and stabilization of soil aggregates. Thus, this study revealed how soil microbial competition or cooperation responded to different fertilization regimes at aggregate scales, and provided evidence for the stimulation of soil stability.

## Introduction

The soil is the most heterogeneous component of the biosphere, with an extremely high differentiation of properties. Soil particles bond strongly to diverse mineral, organic and biotic substrates during the various physical, chemical, and biological processes, consequently forming micro- and macroaggregates (Totsche et al., 2018). The distribution of micro- and macroaggregates causes various physical structures, e.g., different pore sizes and connectivity (Li et al., 2017), water and oxygen availability (Ebrahimi and Or, 2016), and substrate content (Bach et al., 2018), which finally lead to the differential distribution of microorganisms (Vos et al., 2013). Macroaggregates contain large pores, with high pore connectivity and oxygen availability, which favor the growth of filamentous fungi (Rillig et al., 2015). Compared with macroaggregates, microaggregates tend to harbor abundant recalcitrant organic substrates and inhibit fast-growing and less diverse microorganisms (Davinic et al., 2012; Kuzyakov and Blagodatskaya, 2015; Bach et al., 2018). Thus, heterogenous niches in soil aggregates can provide more detailed and comprehensive information than bulk soil.

Stable aggregates provide an optimum habitat for microorganism, and the microorganisms living in these spaces can also affect soil aggregation processes (Rillig et al., 2017). As widespread microorganisms, the interactions between bacteria and fungi are vital drivers of many ecosystem functions and are important for soil health (Frey-Klett et al., 2011; Deveau et al., 2018). For instance, bacteria can secrete biopolymers that act as binding agents for aggregates at a microbial scale (D’Souza et al., 2018), while fungal hyphal networks can span air pores and transport water and nutrients (Peay et al., 2016). The indirect bacterial and fungal interactions by modifying their microenvironment can also affected their patterns positively or negatively (Deveau et al., 2018). For instance, fungal hyphae efficiently colonize heterogeneous environmental habitats, creating new microhabitats and thereby offering various services which can promote or inhibit the growth of bacteria (de Boer, 2017; Wagg et al., 2019). Hence, studying bacterial and fungal interactions in soil aggregates helps us determine the mechanism of soil stability.

The inputs of organic substrates induce faster biochemical process rates and much more intensive microbial interactions in small zones, which are so-called hotspots, e.g., the soil aggregate surface (Kuzyakov and Blagodatskaya, 2015). Hence, as one of the critical practices of adding extra resources, fertilization regimes exert strong impacts on soil aggregate distribution and microbial diversity, structure, as well as microbial interactions (Trivedi et al., 2015; Zheng et al., 2018; Luan et al., 2021; Zhang et al., 2021). Inorganic fertilization reduces microbial richness, but increases the evenness of oligotrophic organisms (Hartman et al., 2015) and the incorporation of C into macroaggregates (Zhang et al., 2015), while organic amendment enhances the relative abundance of copiotrophs through an increase in the availability of labile carbon sources (Trivedi et al., 2015) and increases the competition between fungi and bacteria (Zheng et al., 2018). However, research on the interactions between bacteria and fungi in different aggregates under different fertilization regimes remains limited.

Microbial co-occurrence networks are widely used to explore the interactions between microbiomes, visualized the response patterns of different taxonomic groups to agronomic practices, and predict the hub species in regulating microbial communities (Banerjee et al., 2018; Hartman et al., 2018; Ma et al., 2020). Recent advances of high-throughput sequencing provide a strong tool to predict microbial co-occurrence patterns in different environment. Indicator species analysis with combinations of site groups, developed by de Cáceres et al. (2010), allows the characterization of the qualitative environmental preferences of the target species (i.e., hub species), and identifies indicators of particular groups of environmental sites (i.e., different soil aggregates). Therefore, the analysis of co-occurrence networks combined with indicator species analysis offered a better understanding of hub species in different soil aggregates.

Based on these findings, we investigated how fertilization regimes affected soil bacterial and fungal communities within aggregates by network analysis based on a 38-year experiment, and determined the hub species in response to soil aggregation. We aimed to determine the following: (1) Do microbial communities in various soil aggregates differ in their responses to fertilization regimes? (2) If so, what the differences of co-occurrence patterns of soil aggregate-associated microbes between different fertilization regimes? (3) Which microbes are the indicator taxa for particular aggregates?

## Materials and Methods

### Experiment Design and Soil Sampling

A 39-year field experiment in a summer-season rice (*Oryza sativa* L.) and winter-season wheat (*Triticum aestivum* L.) rotation system was carried out since 1981 at the Ezhou, Hubei Province, China (114.7°E, 30.4°N). The average yearly rainfall at the site is 1314.0 mm, and the average yearly temperature is 14.2°C. The soil is derived from yellow-brown paddy soil, classified as Udalfs. In this field, there is a complete randomized design, with different fertilization regimes and three replicates. Each plot was set as 5 m × 8 m. The fertilization regimes in this study we used contained: (1) no fertilizer, CK, (2) inorganic nitrogen (N), phosphate (P), and potassium (K) fertilizers, NPK and (3) NPK combined with organic manure, NPKM. We applied urea (180 kg N ha^–1^), superphosphate (90 kg phosphorus pentoxide kg P ha^–1^) and potassium chloride (90 kg potassium dioxide ha^–1^ y) as N, P, and K fertilizers, respectively. The properties of composted swine manure were: 69% moisture, 262.18 g kg^–1^ soil organic carbon, 15.1 g kg^–1^ TN, 20.8 g kg^–1^ phosphorus pentoxide, and 13.6 g kg^–1^ potassium dioxide (11,250 kg ha^–1^ half year). All P, K and organic fertilizers were applied annually before cultivation, and the rates of N applied in the initial, jointing and tillering stages was 2:2:1.

### Soil Sampling and Aggregate Analysis

Intact soil samples were collected at October 2019, and the soil surface plant detritus was removed. Before sampling, we chose a relatively neat area, dug profiles with a shovel, and transferred soil blocks into clear and sterile plastic boxes. Five blocks (20 cm length × 10 cm width) from the 0–20 cm layer were collected from each plot. The plastic boxes were transported to the laboratory without damage from external forces and stored at 4°C until further preparation.

Before aggregate fractionation, soil blocks were separated gently by hand through a 5 mm mesh. Rock, roots and other plant detritus were removed. Next, the soil aggregate fractions [i.e., large macroaggregates (LM) > 2 mm, macroaggregates (MA) 0.25–2 mm, microaggregates (MI) 0.053–0.25 mm, and silt and clay (SC) < 0.053 mm] were obtained with the wet-sieving method modified by Kaiser et al. (2015). Briefly, a fresh subsample (100 g dry weight) was placed on the top mesh of a 2 mm sieve and gently submerged in deionized water for 5 min. Then, aggregates separation was performed manually by moving the sieve up and down 5 cm for 2 min at a rate of 25 times min^–1^. Soil aggregates from the same size were mixed and divided into two subsamples. In total, 72 samples were collected [three fertilization regimes (CK, NPK, NPKM) × 3 replicates × 2 subsamples × 4 soil aggregates]. All aggregates were stored at -80°C for microorganism analysis.

### DNA Extracting and Sequencing

DNA was extracted from 500 mg of soil using FASTDNA Spin Kit (MP Biomedical, Santa Ana, CA, United States) according to the manufacture’s protocols. The DNA concentration and quality were checked by NanoDrop (Thermo NanoDrop 2000, Delaware, United States). For bacteria, the V3-V4 hypervariable regions of the 16S rRNA gene were targeted using primers 515F (GTGCCAGCMGCCGCGGTAA) and 806R (GGACTACVSGGGTATCTAAT); for fungi, the ITS1 region was amplified using ITS1F (CTTGGTCATTTAGAGGAAGTAA) and ITS2R (GCTGCGTTCTTCATCGATGC). Sequencing was performed on the Illumina MiSeq platform.

Sequenced paired-end reads were joined using FLASH^1^, quality filtered using Fastp tools^2^. Briefly, sequences were joined (overlapping pairs) and grouped by samples following the barcodes and then barcodes were removed. Then, sequences < 50 bp or with ambiguous base calls were removed. Reads were denoised by unoise3 and any chimeric sequences were removed using the USEARCH tool^3^ based on silva_16s_v123. Sequences were then split into operational taxonomic units (OTUs) at 97% similarity using the UPARSE pipeline in USEARCH. Plastid and non-bacteria were removed by USEARCH based on silva_16s_v123. The involved quality filtering and 97% clustering of the ITS1 region as indicated above for the 16S processing, using the UNITE database (utax_reference_dataset_all_02.02.2019) for chimera removal, taxonomic identification and non-fungi removal of representative OTUs. Both bacterial and fungal OTU tables were resampled into a minimum reads per sample. The raw sequencing data were deposited in the NCBI Sequence Read Archive (SRA^4^) with accession No. PRJNA784426.

### Statistical Analysis

All data were managed by Microsoft Excel 2016 (Microsoft Corporation, Washington, DC, United States), all statistical analyses were conducted in R (version 3.6.1), and some figures in this manuscript were revised by Adobe Illustrator CS6 (Adobe Systems Incorporated, California, CA, United States). We used the R script reported by Hartman et al. (2018) for reference. The R script and required files are provided in the additional file and the flowchart of our data analysis was provided in Supplementary Figure 1.

To reduce the false positive results causing by low reads, filtered OTU tables were normalized by the trimmed mean of *M*-values (TMM) method using the BioConductor function in the “edgeR” package (Robinson et al., 2010) and expressed the normalized counts as relative abundance counts per million (CPM). Alpha diversity was determined by USEARCH on the filtered OTU tables; differences between the fertilization regimes and soil aggregates were tested with permutational analysis of variance (PERMANOVA) using the *adonis* function in the ‘‘vegan’’ package with 10^4^ permutations^5^. Unconstrained principal coordinates analysis (PCoA) was conducted to compare the difference in the bacterial and fungal communities between the fertilization regimes and soil aggregates based on Bray-Curtis dissimilarities with the “phyloseq” package (McMurdie and Holmes, 2013). The constrained analysis of principal coordinates (CAP) was used to test the effects caused by soil aggregation using the ‘‘phyloseq’’ package and *betadisp* function in the ‘‘vegan’’ package. Pairwise comparisons between soil aggregates were also performed using the *pairwise.perm.manova* function in the ‘‘RAVideMemoire’’ package^6^. The taxonomic patterns of bacterial and fungal communities were based on the average of relative abundances (RAs) from six replicates.

To identify OTUs that were correlated with community separation between fertilization regimes and soil aggregates, we conducted differential OTU abundance analysis by fitting a generalized linear model with normalized values and testing for differential abundance using a likelihood ratio test (LRT) in “edgeR” package with a false discovery rate (FDR) corrected value of *p* < 0.05. The relative changes between groups were log transformed using the ‘‘dplyr’’^7^, “limma” (Ritchie et al., 2015), and “edgeR” packages. To quantify the specific effects of fertilization regimes and soil aggregation on bacterial and fungal community compositions, we conducted a variance partial analysis using the *varpart* function in the “vegan” package. To identify the soil aggregation-sensitive OTUs (asOTUs, which are sensitive to soil aggregation), we used the correlation-based indicator species analysis with the “indicspecies” package (de Cáceres et al., 2010) to calculated the point-biserial correlation coefficient (*r*) of an OTU’s positive association with a specific aggregate or shared by different fractions. Then, we tested for differential OTU abundances between specific or shared aggregates in different fertilization regimes using LRT in the “edgeR” package with FDR correction. Therefore, OTUs jointly determined by indicator species analysis and LRT with an FDR corrected value at *p* < 0.05 were regarded as soil asOTUs. The asOTUs obtained from indicator species analysis was visualized using bipartite networks with Fruchterman-Reingold layout in the ‘‘igraph’’ package^8^.

Co-occurrence networks were conducted by the TMM-normalized CPM counts with significant Spearman correlations between OTUs (|ρ| > 0.7 and *p* < 0.05). For individual bacterial and fungal networks in different fertilization regimes, the descriptive and topological network characteristics were calculated, which included the total number of network nodes and edges, the number of positive (ρ > 0.7, *p* < 0.05) and negative (ρ < -0.7, *p* < 0.05) correlated edges, and the degrees of co-occurrence (number of direct correlations to a node). For combined bacterial and fungal co-occurrence networks, significant Spearman correlations between all pairs of bacterial OTUs and fungal OTUs were performed, and the network characteristics mentioned above were also calculated. For in-depth analysis, we sub-structured networks with nodes possessing a high density of edges, which identified modules. These modules were identified by the greedy optimization of the modularity algorithm in the ‘‘igraph’’ package. The keystone OTUs were defined as the top 1% of the node degree values of each network (Berry and Widder, 2014), and their RAs were visualized in the ‘‘pheatmap’’ package^9^. The distribution of microbial interactions was visualized in the waffle chart using the ‘‘waffle,’’^10^ ‘‘ggplot2,’’^11^ and ‘‘dplyr’’ packages^12^. Potential microbial phenotypes were predicted with BugBase (Ward et al., 2017).

## Results

### Soil Bacteria and Fungi in Different Fertilization Regimes

**FIGURE 1:**
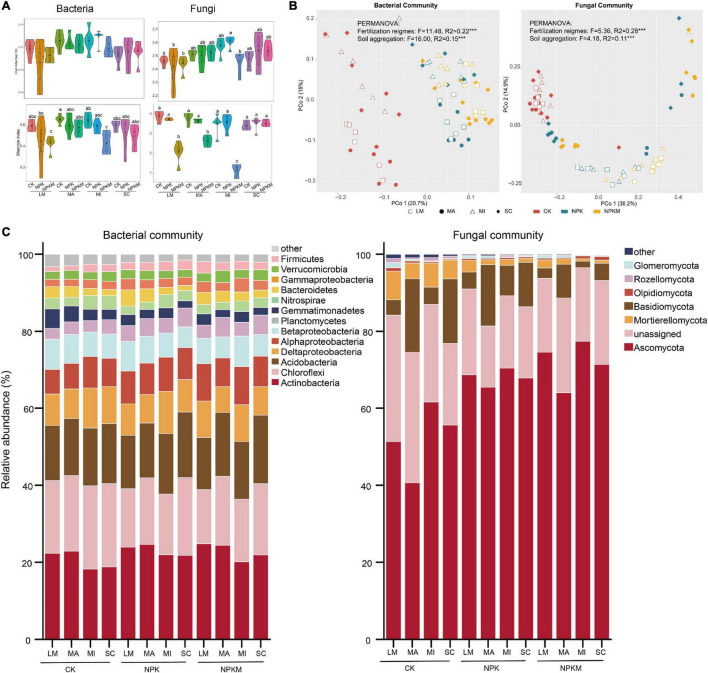
Bacterial and fungal community compositions in different aggregate fractions under different fertilization regimes. **(A)** Alpha diversity [Chao (upper) and Shannon (below)] of soil bacteria (left) and fungi (right) community. The data of Shannon index was log-transformed. Different letters indicated significant difference at *p* < 0.05 (FDR corrected). **(B)** Unconstrained PCoA ordinations of bacteria (left) and fungi (right). Fertilization regimes are colored and aggregates are shaped by sample type. **(C)** Taxonomic profiles of bacteria and fungi communities at phylum level. Bacteria and fungi phyla with relative abundances lower than 1% were summarized with ‘other’ (Bray–Curtis). LM, large macroaggregates; MA, macroaggregates; MI, microaggregates; SC, silt and clay.

Measures of α-diversity revealed that fertilization regimes and soil aggregation exerted significant impacts on bacterial and fungal diversity estimated by the Shannon index (fertilization regimes bacteria *p* = 0.001, fungi *p* = 0.001; soil aggregation bacteria *p* = 0.027, fungi *p* = 0.015), while only soil aggregation significantly influenced bacterial and fungal richness estimated by Chao 1 (bacteria *p* = 0.002, fungi *p* = 0.003; Figure 1A and Supplementary Table 1). For the fertilization regimes, compared with CK, NPKM treatment significantly decreased soil bacterial and fungal diversity. Compared within soil aggregates, soil bacterial diversity and richness were highest in MA and MI, while fungal richness was highest in MI. Among all treatments, soil fungal diversity was highest in MI in NPK treatment, while bacterial richness were lowest in LM and MI in NPKM.

To visualize and quantify the differences between microbial communities, we conducted unconstrained PCoA based on Bray–Curtis dissimilarities and CAP (Figure 1B, Supplementary Figure 2, Supplementary Table 1, and Supplementary Datasets 2, 3). Result combined with PERMANOVA and pairwise tests indicated that both fertilization regimes (*p* < 0.001) and soil aggregation (*p* < 0.001) exerted significant impacts on bacterial and fungal community compositions. Soil microbes in LM and MA spread well to the microbes in MI and SC, except fungi in NPK. A significant difference in dispersion test revealed that differences in bacterial and fungal communities in the NPK and NPKM treatments were also driven by soil aggregation, except fungi in CK (Supplementary Table 2 and Supplementary Dataset 4). In summary, soil bacterial and fungal communities were driven by both fertilization regimes and soil aggregation.

Taxonomies of bacterial and fungal communities at the phylum level are visualized in Figure 1C. The bacteria in all samples were dominated by the members of Actinobacteria (19.8%), Chloroflexi (18.1%), Acidobacteria (14.6%), Deltaproteobacteria (8.2%), Alphaproteobacteria (7.5%), Betaproteobacteria (6.2%), and Nitrospira (3.8%) (Figure 1C). Among the fertilization regimes, the RAs of Actinobacteria (21.4%), Alphaproteobacteria (8.2%), Planctomycetes (4.0%), Bacteroidetes (2.4%), Gammaproteobacteria (2.6%), and Firmicutes (2.2%) were highest in NPKM. Among soil aggregates, the RAs of Deltaproteobacteria (10.1%), Betaproteobacteria (7.0%), and Gemmatimonadetes (3.5%) were highest in LM; the RAs of Chloroflexi (20.7%), Acidobacteria (16.1%), and Verrucomicrobia (2.9%) were highest in SC; and the RA of Actinobacteria (21.4%) was highest in MI. For fungi, the dominant members were affiliated with the phyla Ascomycota (56.8%) and Mortierellomycota (12.0%). Among fertilization regimes, the RA of Ascomycota (71.8%) was highest in NPKM, and the RA of Mortierellomycota (20.5%) was highest in NPK. Among soil aggregates, the RA of Ascomycota (66.4%) was highest in MI, but lowest in SC, the RA of Mortierellomycota (20.9%) was highest in SC, and the RAs of Basidiomycota (6.8%), Chytridiomycota (0.6%), and Glomeromycota (0.7%) were the highest in LM.

According to phenotype prediction based on BugBase, we found that both fertilization regimes and soil aggregation significantly influenced microbial phenotypes (Supplementary Figure 2 and Supplementary Dataset 2). For example, lower RAs of facultatively anaerobic, Gram-negative and Gram-positive bacteria, but higher RAs of aerobic bacteria and highest concentration of mobile elements were occurred in NPKM. Compared within the aggregates, the microhabitat in MI favored the growth of facultatively anaerobic- and Gram-positive bacteria, stimulated biofilm formation, and enhanced stress tolerance.

### Soil Bacterial and Fungal Communities Respond to Soil Aggregation

**FIGURE 2:**
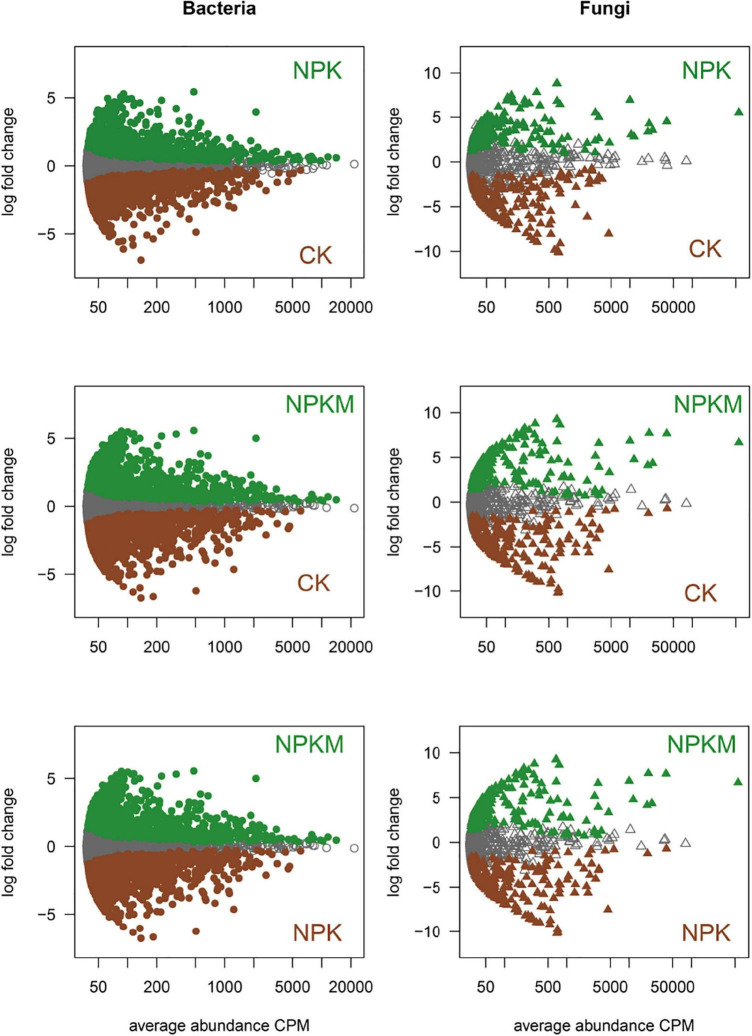
Specific sets of soil microbes under different fertilization regimes. MA plots displayed the abundance patterns of soil bacteria and fungi under CK, NPK, and NPKM treatments. *X*-axis is average OTU abundance (as counts per million, CPM), and Y-axis is log2–fold change. Non-differentially abundant OTUs are in gray, and fertilization regimes-specific OTUs are colored in darkgreen and brown, respectively (likelihood ratio test, *p* < 0.05, FDR corrected).

Different fertilization regimes and soil aggregation shaped the microbial community and harbored specific microbial sets (Figure 2 and Supplementary Figure 3). Based on OTU counts from CK as a control and an adjusted *p*-value cutoff of 0.01, there were 836 and 1150 bacterial OTUs, and 155 and 171 fungal OTUs that were significantly enriched in the NPK and NPKM treatments, respectively. There were also 944 bacterial OTUs and 109 fungal OTUs significantly enriched in NPKM compared with NPK. For an in-depth analysis of the soil aggregation effects on soil microbial communities, we explored the bacterial and fungal OTUs enriched and depleted in different aggregates under different fertilization regimes. The fungal community in different aggregates was similar between the NPK and NPKM treatments, as indicated by the minority in the MA plot. Compared to soil microbes in MI and SC, bacterial OTUs were more enriched and depleted in MA and LM, while fungal OTUs were significantly enriched in MI.

**FIGURE 3:**
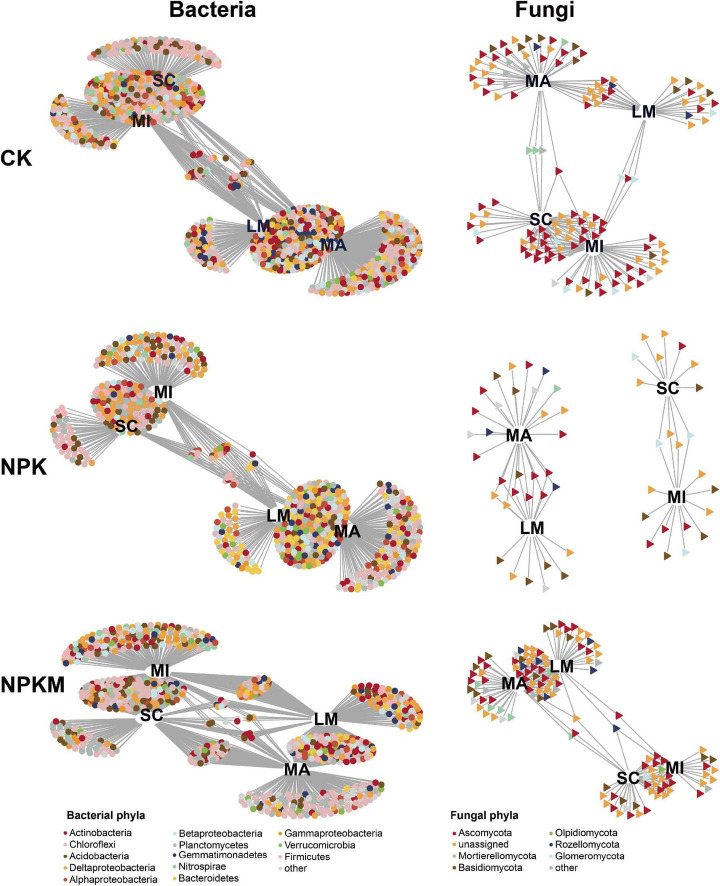
Bipartite networks display soil aggregation specific bacterial and fungal OTUs in different fertilization regimes determined by indicator species analysis. Circles represent bacteria and triangle represent fungi. OTUs are colored according to the phylum assignment. See Figure 1.

The result of VPA in quantifying the effects on soil microbes, revealed that fertilization regimes exerted a larger effect on soil bacterial (19.8%) and fungal (29.0%) communities than soil aggregation (bacteria 11.4%, fungi 6.0%) (Supplementary Figure 4). We defined the OTUs that were supported by indicator species analysis and likelihood ratio tests as soil aggregation sensitive OTUs (asOTUs, Supplementary Figure 4 and Supplementary Dataset 5) and visualized the result in a bipartite network (Figure 3). In all, there were 859, 467, and 640 bacterial asOTUs, and 109, 38, and 113 fungal asOTUs in the CK, NPK and NPKM treatments, respectively. Thus, bacterial asOTUs accounted for 34.7, 20.6, and 22.8% of the total bacterial OTUs, and fungal asOTUs accounted for 34.7, 20.6, and 15.6% of the total fungal OTUs in the CK, NPK, and NPKM treatments, respectively. Slight overlap was found between fungal asOTUs among different fertilization regimes compared with bacterial asOTUs (Supplementary Figure 4), which was consistent with the VPA result that fertilization regimes impacted the fungal community more than the bacterial community (Supplementary Figure 4). The bacterial asOTUs were mainly consisted of Proteobacteria, Chloroflexi, and Acidobacteria, and the fungal asOTUs were primarily affiliated to Ascomycota and Mortierellomycota which were significantly enriched in all aggregates (Supplementary Dataset 5). Furthermore, soil aggregation exerted significant effects on taxonomic patterns.

The bipartite networks clearly showed that both bacteria and fungi in LM and MA were strongly correlated with each other and shared many OTUs, but were separated well from the microbiomes in MI and SC (Figure 3), which confirmed the results of PCoA and CPA. In addition, more bacterial and fungal taxa were shared among different aggregates in NPKM than taxa in NPK (Supplementary Dataset 6), which suggested that microorganisms in NPKM had stronger correlations within aggregates. Moreover, the abundances of both bacterial and fungal asOTUs in MA were significantly higher compared within aggregates, except for the higher bacterial asOTUs in MI in the NPKM treatment. Due to their taxonomic patterns, most bacterial asOTUs in MA and SC belonged to the phylum Chloroflexi (25.1–42.2%), and asOTUs in LM and MI belonged to the phylum Proteobacteria (18.6–43.2%), especially the class Deltaproteobacteria (10.2–28.9%). Most fungal asOTUs in all fertilization regimes were affiliated with the phyla Ascomycota (12.5–50.0%) and Basidiomycota (7.4–57.1%). Therefore, although the majority of communities were shared between soil aggregates, both fertilization regimes and soil aggregation caused specific microbial sets.

### Soil Microbial Co-occurrence Patterns Respond to Soil Aggregation

**FIGURE 4:**
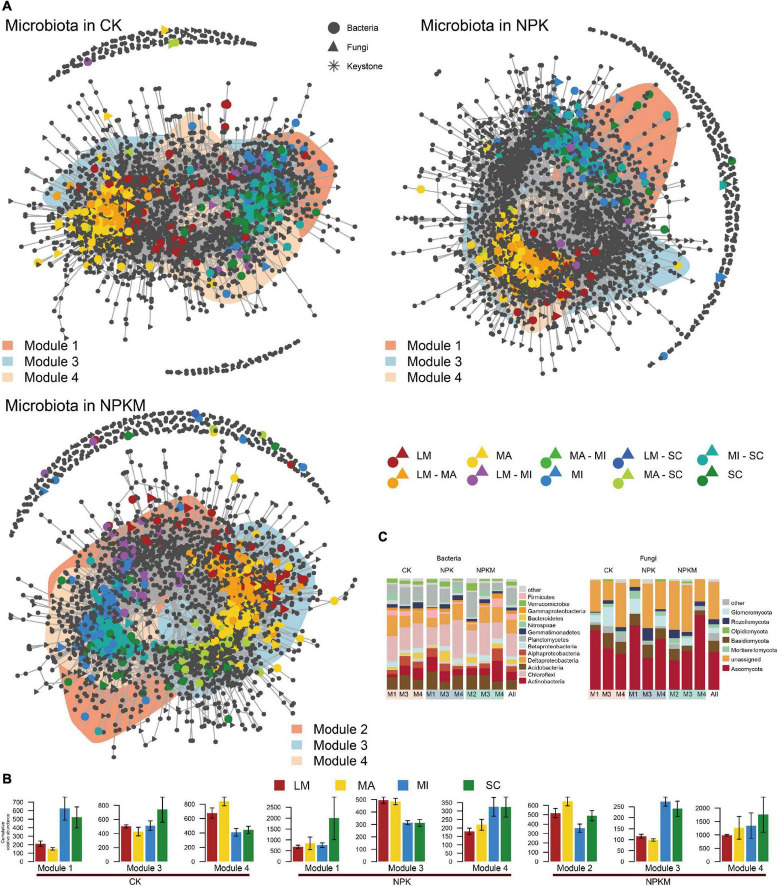
Co-occurrence patterns of soil aggregation sensitive OTUs under different fertilization regimes. **(A)** Co-occurrence networks visualizing significant correlations (*p* < 0.001; indicated with gray lines) between soil bacterial and fungal OTUs under different fertilization regimes. Circles indicate bacteria, triangles fungi and asterisks indicate keystone OTUs. Soil aggregation sensitive OTUs are colored and shade area are represented the network modules (the OTUs in each module are defined in Supplementary Figure 4). **(B)** Cumulative relative abundance (as counts per million, CPM; *y*-axis in ×1000) of all soil aggregation sensitive bacteria and fungi in each module under different fertilization regimes. **(C)** Qualitative taxonomic composition of soil aggregation sensitive modules is reported as proportional OTUs numbers per phylum and compared to the overall taxonomic distribution in the entire dataset (column ‘all’). See Figure 1.

To explore how fertilization regimes and soil aggregation impact soil microbial co-occurrence patterns, we constructed bacterial and fungal networks (Figure 4 and Supplementary Figure 6). We first built separate networks for the soil bacterial and fungal communities in different fertilization regimes and determined their properties (Supplementary Figure 6 and Supplementary Dataset 7). More complex bacterial networks and higher positive correlations among OTUs were present in all networks under the different fertilization regimes (Supplementary Table 3). Consistent with the α-diversity result, the soil bacterial and fungal network comprised the highest number of significantly co-occurring OTUs in CK, while the NPKM treatment decreased the number of co-occurring bacterial and fungal OTUs. The average network connectivity of microbial communities was lower, and the number of asOTUs was less in NPK and NPKM than those in CK. The asOTUs in all networks were divided into two groups—one that primarily originated from LM and MA, and the other mostly from MI and SC. Furthermore, we classified these asOTUs into taxonomic patterns and found that the distribution of specific sets responded to soil aggregation (Supplementary Datasets 8–13). For the bacterial community in all networks, most bacterial asOTUs were affiliated with the phyla Proteobacteria (CK 27.1%, NPK 26.6%, NPKM 23.9%) and Chloroflexi (CK 23.1%, NPK 19.4%, NPKM 25.3%). Among the Proteobacteria class, Deltaproteobacteria showed the highest RA in all fertilization regimes (CK 15.2%, NPK 13.5%, NPKM 11.5%). For fungi, except unassigned fungi, the phylum Ascomycota occupied a major part of the fungal networks (CK 47.0%, NPK 30.0%, NPKM 34.4%). In all these nodes, most bacterial asOTUs in all these networks belonged to OTUs specific in MA (CK 16.2%, NPK 14.4%, NPKM 14.8%) in all fertilization regimes. Meanwhile, most fungal asOTUs in CK, NPK, and NPKM were found in MA (25.7%), MI (26.5%), and SC (25.1%), respectively.

Then, we contrasted the bacterial and fungal co-occurrence network under different fertilization regimes (Figure 4). Consistent with separate co-occurrence network, the inter-kingdom microbial patterns also responded sensitively to soil aggregation. Compared to CK, the co-occurrence network in the NPK and NPKM treatments was less complicated, especially less connections between bacterial and fungal communities (Table 1). Based on the analysis of correlations between and within bacterial and fungal asOTUs, we found that the interactions between and within bacteria (B) and fungi (F) were mostly positive [CK Bacteria–Bacteria (B–B) 77.6%, Bacteria–Fungi (B–F) 68.5%, Fungi–Fungi (F–F) 92.5%; NPK B–B 66.6%, B–F 68.8%, F–F 89.4%; NPKM B–B 45.5%, B–F 56.3%, F–F 79.2%] (Supplementary Dataset 14). The interactions within specific aggregates were all positive, but interactions between aggregates were mostly negative. Furthermore, the interactions within and between bacteria and fungi in NPK and NPKM were significantly less in LM, MA, and SC, but higher in MI compared to interactions in CK. Specifically, unlike the interactions in NPK, their positive correlation within bacteria was enriched, but less correlation within fungi was found in MI in NPKM. Moreover, for in-depth taxonomic analysis (Supplementary Figure 10), we found that most taxa in these interactions were affiliated with Chloroflexi, Deltaproteobacteria, and Ascomycota. For instance, the interactions within bacteria in MA were dominated by Chloroflexi, and the interactions were more diverse in MI, such as correlations between Deltaproteobacteria and other phyla, e.g., Chloroflexi, Acidobacteria, Actinobacteria, and Nitrospirae. For fungi, the interactions between Mortierellomycota, Basidiomycota and Ascomycota were more diverse in MA in NPKM. With respect to the interactions between bacteria and fungi in NPKM, the correlations between Basidiomycota and Chloroflexi were dominant in MA and MI.

**TABLE 1:** Properties of bacterial and fungal co-occurrence networks in different fertilization regimes.

	OTUs		Connections						Connectivity	Keystones		asOTUs	
	B	F	B-B		F-F		B-F			B	F	B	F
			+	–	+	–	+	–					
CK	2785	444	38532	11107	3280	265	9232	4245	31.62	31	1	822	108
NPK	2680	343	17238	8654	303	36	2146	975	13.02	30	1	446	35
NPKM	2562	326	6893	8264	494	130	2673	2072	13.83	27	2	605	105

*B, bacteria; F, fungi; +, positive; –, negative.*

To identify microbial keystones, we separated all OTUs into modules and analyzed the three major modules in the top 20 modules, which occupied all OTUs over 85% (Supplementary Figure 7 and Supplementary Dataset 15). Although these three modules contained most of the asOTUs in each network, these modules did not separate well from each other (Figure 4A), which indicated close interconnections between soil aggregates. Consistent with the results of bipartite networks, asOTUs in LM were closely related to OTUs in MA, but dispersed well from the two other fractions (Figure 4B). For taxonomic pattern analysis (Figure 4C), the bacterial phyla Proteobacteria (especially Deltaproteobacteria) and Chloroflexi, and fungal phylum Ascomycota comprised the major parts of the co-occurrence networks. Based on an inspection of aggregates, we found that all OTUs from LM and MA were positively correlated with each other, but negatively correlated with OTUs from MI and SC, and vice versa (Supplementary Datasets 14, 16). Moreover, interactions within MA and MI were more intensive than the other two fractions. For taxonomic pattern analysis, OTUs from the phylum Chloroflexi were dominant within bacterial OTUs in all aggregates, while most taxa in MI were belonged to the phylum Acidobacteria and Proteobacteria (especially Deltaproteobacteria) in NPK and NPKM treatments, respectively. Except for unassigned OTUs, most fungal OTUs were positively correlated and mostly belonged to the phylum Ascomycota, which were abundant in the MA and MI fractions.

**FIGURE 5:**
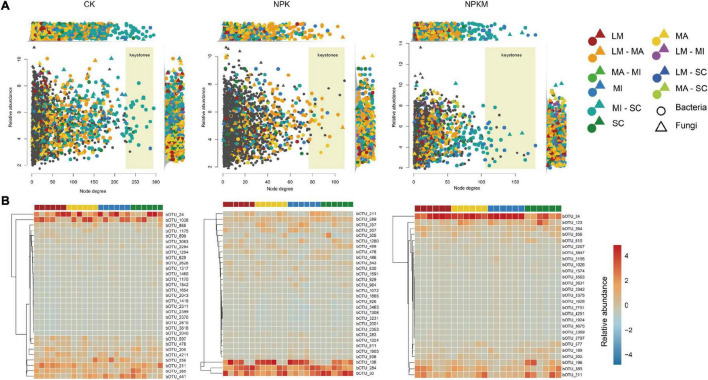
Identified soil aggregation-sensitive OTUs (asOTUs) and keystones. **(A)** Degree of co-occurrence and abundances of asOTUs. Relative abundances (as counts per million, CPM) of all OTUs from co-occurrence networks (Figure 4) were plotted as a function of their degrees of co-occurrence. OUTs were colored by their associations to soil aggregate fractions, and circle and triangle represented bacteria and fungi, respectively. OUTs in the yellow background were identified as keystones. Top panels revealed the distribution of node degrees and side panels revealed the relative abundances of all asOTUs. **(B)** Relative abundances of bacterial keystones determined by co-occurrence network. Keystones was centroid clustered by Euclidean distance. The deeper the color is, the higher relative abundance of keystones is. B, bacteria; F, fungi, others see Figure 1.

The asOTUs were identified among low counts and high RAs among microbes in different soil aggregates in different fertilization regimes (Figure 5A and Supplementary Dataset 17), which revealed that keystones in networks appeared to reflect differences in fertilization regimes and soil aggregation. There were 822 bacterial and 108 fungal asOTUs in the CK treatment, 446 bacterial and 35 fungal asOTUs in the NPK treatment, and 605 bacterial and 105 fungal asOTUs in the NPKM treatment (Table 1). In the NPKM and CK treatments, asOTUs specific to MI and shared by MI and SC exhibited higher node degrees than asOTUs in other fractions. However, keystones in the NPK treatment originated from OTUs that were specific to MA or shared by MA and LM fractions. For the analysis of asOTU taxonomic patterns (Supplementary Figure 8), we found that the fungal phyla Acidobacteria (CK 14.8%, NPK 20.8%, NPKM 15.0%), Chloroflexi (CK 16.2%, NPK 17.1%, NPKM 22.1%), Ascomycota (CK 34.8%, NPK 56.9%, NPKM 42.8%), and Mortierellomycota (CK 20.3%, NPK 34.8%, NPKM 22.2%) were abundant in all fertilization regimes.

Soil aggregation also exerted significant effects on the distribution of taxonomic patterns. The phylum Ascomycota was most abundant in LM and less abundant in SC, while the phylum Mortiellomycota showed the opposite trend (Figure 5B, Supplementary Table 3, and Supplementary Dataset 18). In CK, the keystone nodes from the Gemmatimonadetes (family Gemmatimonadaceae bOTU_478), the Proteobacteria (order sva0485 bOTU_211), and the Ascomycota (family fOTU_411) had higher RAs in LM. In NPK, the keystone nodes from the Chloroflexi (the genus *Anaerolinea* bOTU_1905, bOTU_3231; the genus *Chloronema* bOTU_1072), the Actinobacteria (the genus *Nocardioides* bOTU_283), the Betaproteobacteria (bOTU_357), the Alphaproteobacteria (the genus *Pedomicrobium* bOTU_811), and the Gammaproteobacteria (the genus *Lysobacter* bOTU_929) had higher RAs in MI; the keystone nodes from Bacteroidetes (the genus *Flavisolibacter* bOTU_136), the Deltaproteobacteria (bOTU_926), and the Nitrospirae (the family Nitrospiraceae bOTU_2001) had higher RAs in LM; and the RAs of keystone nodes from Bacteroidetes (bOTU_1280) and the Deltaproteobacteria (the family Syntrophobacteraceae bOTU_543, the genus *Geoalkalibacter* bOTU_1865) were higher in SC. In NPKM, the RAs of keystone nodes from the Deltaproteobacteria (bOTU_856), the Acidobacteria (the family AKIW659 bOTU_196), and the Nitrospirae (bOTU_610) were higher in LM; and the keystone nodes from the Acidobacteria (bOTU_264, bOTU_1026, the genus *Blastocaella* bOTU_2342), the Chloroflexi (the family Anaerolineaceae bOTU_2267, the genus *Roseiflexus* bOTU_3675), the Actinobacteria (the genus *Rubrobacter* bOTU_3563), and the Nitrospirae (the genus *Nitrospira* bOTU_3847) had higher RAs in MA.

## Discussion

### Different Responses of Soil Microbiomes to Different Fertilization Regimes

We confirmed that fertilization regimes altered the microbial communities’ diversity and compositions (Tsiafouli et al., 2015), but exerted little impact on microbial richness. This is consistent with previous findings that species richness responded less to environmental factors than species composition (Brown et al., 2017) and the changes of some taxonomic groups may be compensated by changes in others (Hartman and Widmer, 2006). Depletion of bacterial and fungal diversity in the NPKM treatment indicates that the organic fertilization created a copiotrophic environment and decreased the competition among microbes, primarily due to the abundant nutrients offered by organic manure. Higher species richness in an oligotrophic environment also helped to dampen the microbial population fluctuations (Romanuk et al., 2006). The significant dissimilarities between soil microbial communities may have the consequence of fertilizers acting as a microbial resource, increasing organic carbon availability and changing edaphic factors (Geisseler and Scow, 2014; Dong et al., 2021).

Higher microbial diversity could induce more interactions with each other directly or indirectly, such as competition, facilitate or inhabitation, and taxonomic richness also supported greater microbiome complexity and interkingdom associations (Wagg et al., 2019). In present study, we observed highest connectivity in CK, which could be explained by the highest microbial diversity. Compared with taxa in NPK, enrichment of taxa in the phyla Actinobacteria, Proteobacteria (especially Alphaproteobacteria and Gammaproteobacteria), and Ascomycota but depletion of Deltaproteobacteria and Mortierellomycota in NPKM, reflected the differences caused by organic manure. Therefore, most taxa in the phyla Actinobacteria and Proteobacteria are suggested to be copiotrophic organisms, which exhibited rapid growth in the NPKM treatment, where available nutrients were abundant (Fierer et al., 2012; Arcand et al., 2017). Unlike other classes of Proteobacteria, Deltaproteobacteria has the potential oligotrophic ability which supports its dominance in soil without fertilization (Aira et al., 2019). Most taxa in Mortierellomycota are classified as arbuscular mycorrhizal species, which supports their predominant role in a nutrient-poor environment (Spatafora et al., 2016). For the correlations within the microbial kingdom across different soil aggregates, we found that soil bacteria and fungi in aggregates, especially fungi in the NPK treatment, were less correlated compared to relationships in the NPKM treatments, which is consistent with a previous study (Ling et al., 2016). This is generally because of the complicated microhabitat caused by the strong organic-mineral binding (Pronk et al., 2016), and to some degree possibly due to the special taxa brought about by the organic manure itself (Kim and Wells, 2016).

### Sensitive Responses of Microbiomes to Soil Aggregation

Soil aggregation significantly altered microbial diversity and composition, as well as microbial richness, although fertilization regimes exerted more impacts on microbial communities. This can be explained by that the spatial heterogeneity within the aggregates and the increased mutation rates because of elevated stress levels (i.e., nutrient depletion and toxin accumulation) may maintain or increase the microbial diversity to some degree (Rillig et al., 2017). In present study, we found highest bacterial diversity and richness in MA and MI, and highest fungal richness in MI, which is consistent with previous studies (Davinic et al., 2012; Upton et al., 2019). They suggested that compared to larger aggregates, complex organic substrates (e.g., phenols and alkyls), and suitable microhabitat (e.g., proper pore connectivity) in MI showed a strong selective force for bacterial community compositions, and environmental conditions between and within aggregates also resulted in diverse niches that harbored different guilds of microorganisms. However, higher enrichment and depletion of bacterial OTUs in LM than MI and SC cannot be shown by the lower diversity and richness. This is possibly because alternations of microbial community composition might not be accompanied by the changes in diversity and richness owing to the changes in some taxonomic groups (Supp and Ernest, 2014), and the specific relationships between individual OTUs are neglected by the univariate measures of diversity and richness (Wu et al., 2007).

We identified soil aggregation-sensitive asOTUs in all fertilization regimes, and they indicated the clear separation of β-diversity patterns by soil aggregation. For example, both bacterial and fungal asOTUs in all fertilization regimes were mostly from MA. This is because MA contains more organic carbon and higher concentration of less chemically complex and new organic matter inputs (Davinic et al., 2012), with optimal living conditions (e.g., appropriate pore connectivity, water and oxygen availability) for the growth of bacterial and fungal communities (Totsche et al., 2018). Furthermore, for the analysis of taxonomic patterns, most bacterial asOTUs found in the LM and MI in all fertilization regimes belonged to the phylum Proteobacteria, especially Deltaproteobacteria, while asOTUs in MA and SC were mostly from the phylum Chloroflexi. For fungal asOTUs, most OTUs with and without long-term inorganic fertilization in all aggregates were primarily affiliated with the phyla Ascomycota and Basidiomycota, which were the most widespread taxa. Previous studies have shown that some saprotrophic Ascomycota and Basidiomycota are critically important to the formation, stabilization and breakdown of soil aggregates due to their filamentous growth nature and excretion products (Ritz and Yong, 2004; Lehmann and Rillig, 2015).

### Soil Aggregation Effects on Microbial Co-occurrence Across Different Fertilization Regimes

The complex bacterial-fungal interactions, mutualism or antagonism, lead to the critical shifts in microbial community compositions, which are also affected by soil niche and ecological process, e.g., soil aggregation (Ma et al., 2020). In the present study, we found that long-term fertilization decreased the microbial network complexity, possibly because of the decreased microbial interactions between and within bacteria and fungi in LM, MA, and SC. On the contrary, long-term organic fertilization enhanced their intensive interactions in MI and increased their diversity, which had the consequence of high biofilm formation and stress tolerance. A previous study demonstrated that microbes within MI might respond to a small pool of labile C, while a larger pool of recalcitrant C remains protected from decomposition (Paustian et al., 2000), which can stimulate microbial interactions in MI. Most biofilms benefit neighboring microbes and adhere to environmental particles, leading to an increase in resilience against external threats and a high efficiency in decomposing recalcitrant substrates (Nadell et al., 2016; Deveau et al., 2018), which finally stimulate the formation and stabilization of soil aggregates.

Moreover, different types of fertilizers also affected the bacterial–fungal interactions in different niches. Compared to the NPK treatment, NPKM significantly decreased the interactions within bacteria, but increased the interactions between and within fungi in LM and MA. That is because the microhabitat in LM had a large pore size and high pore connectivity, which favor the growth of filamentous fungi (Gupta and Germida, 2015). More complex interactions between Chloroflexi, Deltaproteobacteria, and Ascomycota occupied a predominant position in all aggregates, mostly owing to their widespread occurrence (Peay et al., 2016). Filamentous Chloroflexi, hydrocarbon-degrading Deltaproteobacteria and saprophytic Ascomycota are able to degrade both recalcitrant and labile substrates (de Boer et al., 2005; Davidova et al., 2018; Speirs et al., 2019), which strengthen their critical roles in the process of soil aggregation. NPKM treatment also significantly enhanced the potential cooperation between Deltaproteobacteria and Nitrospirae, which participate in N cycling.

In addition, the bacterial and fungal taxa between LM and MA, MI and SC were positively correlated, whereas taxa between the other two groups were negatively correlated. Negative correlations may originate from a wide range of co-exclusion mechanisms, including direct competition, environmental modification, and differential niche adaptation (Faust et al., 2012). The higher proportion of negative interactions suggested that heterogenous microhabitats increased direct competition, and more biofilms formed in the MI and SC fractions. Therefore, according to the above results, we suggested that the microhabitats of microorganisms could be divided into two groups, larger fractions (LM and MA) and smaller fractions (MI and SC), which is consistent with a previous study (Bach et al., 2018). This finding reflected that soil microorganisms adapted distinctly to larger fractions and smaller fractions as different microhabitats during soil aggregation, e.g., pore connectivity (Yan et al., 2016), water and oxygen availability (Li et al., 2017), and substrate concentration (Huang et al., 2005).

The importance of keystones is intuitive because they are potentially associated with a high number of other species (Berry and Widder, 2014) and exert their influence by selectively modulating accessory microorganisms (Banerjee et al., 2018). Although the microbial co-occurrence network was more complicated in CK, the bacterial keystones were much more under long-term fertilization. In the present study, we found 18 soil aggregation-sensitive keystone taxa in NPK and 17 keystone taxa in NPKM treatment. There into, for keystones in NPK, *Anaerolinea* and *Nocardioides*, the genera from the phyla Chloroflexi and Actinobacteria, respectively, are cells with filaments and anoxygenic photolithotrophic members (Hanada and Pierson, 2006; Liang et al., 2015; Yamada and Sekiguchi, 2018), which are abundant in MI. The genus *Ohtaekwangia*, belonging to the family Cytophagaceae which is mostly aerobic and filament-shaped (Nakagawa, 2015), are also enriched in MI. The genus *Geoalkalibacter* (the class Deltaproteobacteria) was significantly enriched in SC, which is consistent with a previous study (Badalamenti et al., 2013). They demonstrated that *Geoalkalibacter* sp. had the ability to bind complete oxidation of simple molecules to the reduction of mineral oxides, and the intensive mineral-organic binding was one of critical mechanisms of forming and stabilizing soil aggregates; thus, the enrichment of *Geoalkalibacter* in SC helped to stabilize soil aggregates. The genera *Lysobacter* and *Pedomicrobium*, as one of the class Gamma- and Alpha-proteobacteria, respectively, were notably abundant in MA and LM in the present study. Their oxygen requirement for living and filamentous shape assisted their potential function in stabilizing soil aggregates (Poindexter, 2006; Reichenbach, 2006). Niches in LM and MA also provided better microhabitat for the growth of the key species *Flavisolibacter*, which requires an aerobic environment (Rosenberg, 2014). For keystones in NPKM, the genus *Roseiflexus*, as one genera of the phyla Chloroflexi, is an obligate anaerobic member with a filamentous shape, able to degrade cellulose (Gupta et al., 2013), and abundant in SC. Although the genera *Rubrobacter*, *Nitrospira*, and *Blastocaella* function in the process of soil aggregation in NPKM, their low RAs did not show any significant difference in each aggregate fraction.

## Conclusion

The present study provides an overview of bacterial and fungal co-occurrence patterns in different aggregate niches under long-term fertilization. Herein, we found that fertilization regimes exerted much greater effects on soil microbial diversity and communities, especially fungal diversity, than did soil aggregation. Under the condition of long-term cultivation without fertilization, microorganisms utilized soil resources primarily by increasing the microbial network complexity and enhancing the microbial interactions within and between different soil aggregates. In contrast, long-term fertilization decreased the microbial interactions in large macroaggregates, macroaggregates and silt and clay fractions, but increased interactions in microaggregates. In particular, manure addition favored the production of biofilms in microaggregates, and enhanced the stress tolerance, which finally stimulated the formation and stabilization of soil aggregates. In future studies, our findings should be verified in different soil types or habitats.

## Data Availability Statement

The datasets presented in this study can be found in online repositories. The names of the repository/repositories and accession number(s) can be found in the article/Supplementary Material.

## Author Contributions

JC did the sampling work, completed the laboratory experiments, analyzed all the experiments’ results, and wrote the first draft of this manuscript. DS, XW, and JS revised the manuscript. DL helped to maintain the long-term experiment and support the sampling work. WZ and GL guided and proofread the manuscript. All authors contributed to the article and approved the submitted version.

## Conflict of Interest

The authors declare that the research was conducted in the absence of any commercial or financial relationships that could be construed as a potential conflict of interest.

## Publisher’s Note

All claims expressed in this article are solely those of the authors and do not necessarily represent those of their affiliated organizations, or those of the publisher, the editors and the reviewers. Any product that may be evaluated in this article, or claim that may be made by its manufacturer, is not guaranteed or endorsed by the publisher.

## Footnotes

1
https://ccb.jhu.edu/software/FLASH/index.shtml

2
https://github.com/OpenGene/fastp

3
http://www.drive5.com/usearch/manual/alpha_metrics.html

4
http://trace.ncbi.nlm.nih.gov/Traces/sra/sra.cgi

5
https://github.com/vegandevs/vegan/

6
https://CRAN.R-project.org/package=RVAideMemoire

7
https://github.com/tidyverse/dplyr

8
https://igraph.org/r/

9
https://CRAN.R-project.org/package=pheatmap

10
https://github.com/hrbrmstr/waffle

11
https://github.com/tidyverse/ggplot2

12
https://github.com/tidyverse/dplyr
