# Supplementary material for: Soil Aggregation Shaped the Distribution and Interaction of Bacterial-Fungal Community Based on a 38-Year Fertilization Experiment in China
Source: Front Microbiol. 2022 Mar 22;13:824681. doi: 10.3389/fmicb.2022.824681 (PMC8981921; doi:10.3389/fmicb.2022.824681)
Supplement: Supplementary file 2 [file Table_1.docx]

**Table S1** Result of PREMANOVA testing the effects of fertilization regimes and soil aggregation on bacterial and fungal α-diversity

|  | Bacteria | | | | Fungi | | | |
| --- | --- | --- | --- | --- | --- | --- | --- | --- |
|  | Chao1 | | Shannon | | Chao1 | | Shannon | |
|  | Pseudo-F | R^2^ | Pseudo-F | R^2^ | Pseudo-F | R^2^ | Pseudo-F | R^2^ |
| Fertilization | 0.612 | 0.017 | 10.106*** | 0.227 | 0.874 | 0.025 | 24.749*** | 0.418 |
| Aggregate | 6.664** | 0.227 | 3.180* | 0.123 | 4.997** | 0.181 | 3.745* | 0.142 |
| Fertilization*Aggregate | 2.623** | 0.325 | 3.767** | 0.408 | 2.895** | 0.347 | 31.746*** | 0.853 |

Significance **p* < 0.05, ***p* < 0.01, *** *p* < 0.001.

**Table S2** Results of PERMANOVA testing the effects of fertilization regimes and aggregate distribution on bacterial and fungal communities. Results of BETADISP testing for differences in multivariate dispersion between soil aggregate fractions under different fertilization regimes in bacterial and fungal communities.

|  | CK | | | | NPK | | | | | | | NPKM | | | | | | | |  |
| --- | --- | --- | --- | --- | --- | --- | --- | --- | --- | --- | --- | --- | --- | --- | --- | --- | --- | --- | --- | --- |
|  | Bacteria | | Fungi | | Bacteria | | | Fungi | | | | Bacteria | | | | Fungi | | | |  |
|  | pseudo-F | R^2^ | pseudo-F | R^2^ | pseudo-F | | R^2^ | | pseudo-F | | R^2^ | | pseudo-F | | R^2^ | | pseudo-F | | R^2^ | |
| **Aggregation** | **3.30***** | 0.33 | **3.28***** | 0.33 | **2.21***** | 0.25 | | **1.82*** | | 0.21 | | **2.53***** | | 0.27 | | **2.26**** | | 0.27 | |  |
| **Pairwise aggregation comparisons** | | | | | | | | | | | | | | | | | | | |  |
| Large macroaggregate | a | | a | | a | | | a | | | | a | | | | a | | | |  |
| Macroaggregate | a | | b | | a | | | a | | | | b | | | | a | | | |  |
| Microaggregate | b | | c | | b | | | a | | | | c | | | | a | | | |  |
| Silt & clay | b | | c | | b | | | a | | | | b | | | | a | | | |  |
| **Multivariate homogeneity of groups dispersions** | | | | | | | | | | | | | | | | | | | |  |
| Aggregation | 5.79** | | 1.58 | | 3.41* | | | 14.57*** | | | | 3.87* | | | | 16.76*** | | | |  |

Note: Significance **p* < 0.05, ***p* < 0.01, *** *p* < 0.001. Different letters in the pairwise comparisons indicate significant differences at *p* <0.05 (FDR corrected).

**Table S3** Bacterial and fungal keystone OTUs identified in CK, NPK and NPKM treatment documented with taxonomy assignments, OTU IDs, degree of co-occurrence values, and sensitivity to aggregation.

|  | Phylum | Class | Order | Family | Genus | Node | Degree | asOTU |
| --- | --- | --- | --- | --- | --- | --- | --- | --- |
| **Microbial community in CK** | | | | | | | | |
| Bacteria | Gemmatimonadetes | Gemmatimonadetes | Gemmatimonadales | Gemmatimonadaceae | Unassigned | bOTU_478 | 274 | Yes |
|  | Proteobacteria | Deltaproteobacteria | Sva0485 | Unassigned | Unassigned | bOTU_211 | 268 | Yes |
| Fungi | Ascomycota | Orbiliomycetes | Orbiliales | Orbiliaceae | unassigned | fOTU_411 | 269 | No |
| **Microbial community in NPK** | | | | | | | | |
| Bacteria | Chloroflexi | Anaerolineae | Anaerolineales | Anaerolineaceae | Anaerolinea | bOTU_1905 | 102 | Yes |
|  |  |  |  |  |  | bOTU_3231 | 77 | Yes |
|  |  |  |  |  | Unassigned | bOTU_1224 | 85 | Yes |
|  |  |  |  |  |  | bOTU_936 | 79 | Yes |
|  |  | Chloroflexia | Chloroflexales | Chloroflexaceae | Chloronema | bOTU_1072 | 88 | Yes |
|  | Nitrospirae | Nitrospira | Nitrospirales | Nitrospiraceae | Unassigned | bOTU_2001 | 95 | Yes |
|  | Proteobacteria | Betaproteobacteria | SC-I-84 | Unassigned | Unassigned | bOTU_357 | 88 | Yes |
|  |  | Gammaproteobacteria | Xanthomonadales | Xanthomonadaceae | Lysobacter | bOTU_929 | 88 | Yes |
|  |  | Deltaproteobacteria | Syntrophobacterales | Syntrophobacteraceae | Unassigned | bOTU_543 | 82 | Yes |
|  |  |  | Desulfuromonadales | Geobacteraceae | Geoalkalibacter | bOTU_1865 | 81 | Yes |
|  |  |  | Sh765B-TzT-29 | Unassigned | Unassigned | bOTU_926 | 81 | Yes |
|  |  | Alphaproteobacteria | Rhizobiales | Hyphomicrobiaceae | Pedomicrobium | bOTU_811 | 77 | Yes |
|  | Gemmatimonadetes | Gemmatimonadetes | Gemmatimonadales | Gemmatimonadaceae | Unassigned | bOTU_1308 | 93 | Yes |
|  | Armatimonadetes | Unassigned | Unassigned | Unassigned | Unassigned | bOTU_3463 | 87 | Yes |
|  | Bacteroidetes | Bacteroidetes_vadinHA17 | Unassigned | Unassigned | Unassigned | bOTU_1280 | 82 | Yes |
|  |  | Sphingobacteriia | Sphingobacteriales | Chitinophagaceae | Flavisolibacter | bOTU_136 | 79 | Yes |
|  |  | Cytophagia | Cytophagales | Cytophagaceae | Ohtaekwangia | bOTU_207 | 77 | Yes |
|  | Actinobacteria | Actinobacteria | Propionibacteriales | Nocardioidaceae | Nocardioides | bOTU_283 | 78 | Yes |
| Fungi | Rozellomycota | unassigned | unassigned | unassigned | unassigned | fOTU_163 | 107 | Yes |
| **Microbial community in NPKM** | | | | | | | | |
| Bacteria | Chloroflexi | Anaerolineae | Anaerolineales | Anaerolineaceae | Unassigned | bOTU_2369 | 179 | Yes |
|  |  |  |  |  |  | bOTU_2267 | 114 | Yes |
|  |  | Chloroflexia | Chloroflexales | Roseiflexaceae | Roseiflexus | bOTU_3675 | 111 | Yes |
|  | Proteobacteria | Gammaproteobacteria | Chromatiales | Ectothiorhodospiraceae | Unassigned | bOTU_577 | 110 | Yes |
|  |  | Deltaproteobacteria | 43F-1404R | Unassigned | Unassigned | bOTU_856 | 110 | Yes |
|  | Actinobacteria | Rubrobacteria | Rubrobacterales | Rubrobacteriaceae | Rubrobacter | bOTU_3563 | 168 | Yes |
|  | Planctomycetes | Phycisphaerae | Phycisphaerales | Phycisphaeraceae | SM1A02 | bOTU_1924 | 164 | Yes |
|  |  | Planctomycetacia | Planctomycetales | Planctomycetaceae | Unassigned | bOTU_1574 | 122 | Yes |
|  |  | OM190 | Unassigned | Unassigned | Unassigned | bOTU_2155 | 108 | Yes |
|  | Gemmatimonadetes | Gemmatimonadetes | Gemmatimonadales | Gemmatimonadaceae | Unassigned | bOTU_2797 | 133 | Yes |
|  | Nitrospirae | Nitrospira | Nitrospirales | 0319-6A21 | Unassigned | bOTU_610 | 128 | Yes |
|  |  |  |  | Nitrospiraceae | Nitrospira | bOTU_3847 | 114 | Yes |
|  | Acidobacteria | Acidobacteria | Subgroup_4 | Unknown_Family | Blastocatella | bOTU_2342 | 105 | Yes |
|  |  |  | Subgroup_3 | AKIW659 | Unassigned | bOTU_196 | 106 | Yes |
|  |  |  | Subgroup_3 | GOUTB8 | Unassigned | bOTU_264 | 133 | Yes |
|  |  |  | Acidobacteriales | Acidobacteriaceae_[Subgroup_1] | Unassigned | bOTU_4251 | 119 | Yes |
|  |  | Subgroup_22 | Unassigned | Unassigned | Unassigned | bOTU_1026 | 107 | Yes |
| Fungi | unassigned | unassigned | unassigned | unassigned | unassigned | fOTU_326 | 160 | Yes |
|  | Ascomycota | unassigned | unassigned | unassigned | unassigned | fOTU_127 | 142 | Yes |
