# Supplementary material for: Soil Aggregation Shaped the Distribution and Interaction of Bacterial-Fungal Community Based on a 38-Year Fertilization Experiment in China
Source: Front Microbiol. 2022 Mar 22;13:824681. doi: 10.3389/fmicb.2022.824681 (PMC8981921; doi:10.3389/fmicb.2022.824681)
Supplement: Supplementary file 3 [file Presentation_1.zip › Supplementary Figures/Supplementary materials and FigS captions .docx]

**Abbreviations and acronyms mentioned in the manuscript**

**LM** large macroaggregate, **MA** macroaggregate, **MI** microaggregate, **SC** silt and clay fractions, **RA** relative abundance;

**PERMANOVA** permutational analysis of variance;

**PCoA** principal coordinates analysis;

**CAP** the constrained analysis of principal coordinates;

**TMM** the trimmed mean of M-values;

**CPM** counts per million, $\text{CPM}={\text{read count of Gene}\text{i}}/{\text{total}\text{ reads}}\times1,000,000$.

**Fig. S captions**

**Fig.S1** The flowchart of analysis steps for this paper. The figures and tables generated as the output form each step are colored in red. More detailed results can be seen in the supplementary dataset.

**Fig.S2** Constrained analysis of principal coordinates (CAP) of soil bacterial and fungal communities in response to different fertilization regimes and soil aggregates. CAP analysis was constrained by soil aggregation using Bray-Curtis distance, and the explained fraction of the total variance is indicated above the plots (with 95% confidence interval, significance assessed with 10^4^ permutations).

**Fig.S3** Microbial phenotypes prediction based on BugBase. Y-axis represent their relative abundance. Abbreviations: see Fig. 1.

Fig.S4 Separate plots displaying the certain enriched and depleted bacterial and fungal OTUs across different soil aggregates under different fertilization regimes. Each point represents an individual OTU, and the position along the X-axis represents the abundance change compared with other group. Enriched OTUs are colored in green, depleted is in red, and non-significant abundant OTUs are in gray (likelihood ratio test, *p* < 0.05, FDR corrected).

**Fig.S5** Soil aggregation sensitive bacterial and fungal OTUs under different fertilization regimes. (A) Variation of partitioning analysis (VPA) is performed to quantify the contribution of fertilization regimes and soil aggregation to bacterial (left) and fungal (right) community variations. (B)Venn diagrams show the number of OTUs responding to fertilization regimes identified with indicator species analysis (blue) and by edgeR (yellow). OTUs identified by both methods were defined as soil aggregation sensitive OTUs (asOTUs).

**Fig.S6** Mean relative abundances of (counts per million, CPM; log10 scale) of soil aggregation sensitive OTUs identified by indicator species analysis and edgeR (see Fig. S3) across different fertilization regimes for soil bacteria and fungi.

**Fig.S7** Individual network visualizing significant correlations (ρ > 0.7, *p* < 0.001; indicated with grey lines) between OTU pairs in bacterial and fungal communities under different fertilization regimes. Circles and triangles represent bacterial and fungal OTUs, respectively, and their associations were colored to the different soil aggregate fractions (gray lines). General network properties are indicated under each network and include: number of OTUs, number of connections, average number of connections between OTUs (avg. connectivity) and the number of aggregation sensitive (asOTU) in the network.

**Fig.S8** Visualized microbial interactions. (A-C) Interactions within bacteria (A), between bacteria and fungi (B), and within fungi (C) in different aggregate fractions under different fertilization regimes were visualized in waffle chart. Their interactions are obtained from the co-occurrence networks. One square represents one link and is colored according to the phylum assignment.

**Fig.S9** Defining network modules. Number of OTUs in the top 20 modules for the soil microbial co-occurrence networks under different fertilization regimes was plotted. Circles and triangles represent bacterial and fungal OTUs, respectively. OTUs were colored by their association to the different aggregate fractions. Percentages on the X-axis indicate the proportion of aggregation sensitive OTUs present in each module.

**Fig.S10** Average relative abundances of soil aggregation sensitive bacterial and fungal OTUs (as defined in Fig. 4, summarized at phylum level) across soil aggregate fractions in different fertilization regimes.
