# Supplementary figures and images for: Soil Aggregation Shaped the Distribution and Interaction of Bacterial-Fungal Community Based on a 38-Year Fertilization Experiment in China
Source: Front Microbiol. 2022 Mar 22;13:824681. doi: 10.3389/fmicb.2022.824681 (PMC8981921; doi:10.3389/fmicb.2022.824681)

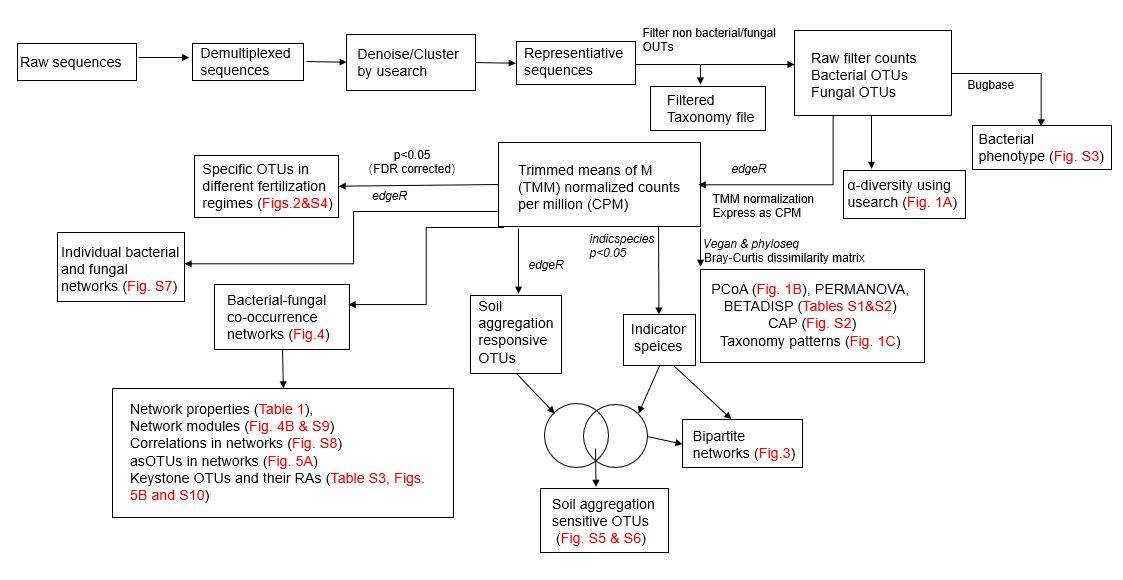

Supplement: Supplementary file 3 [file Presentation_1.zip › Supplementary Figures/FigS1.jpg]

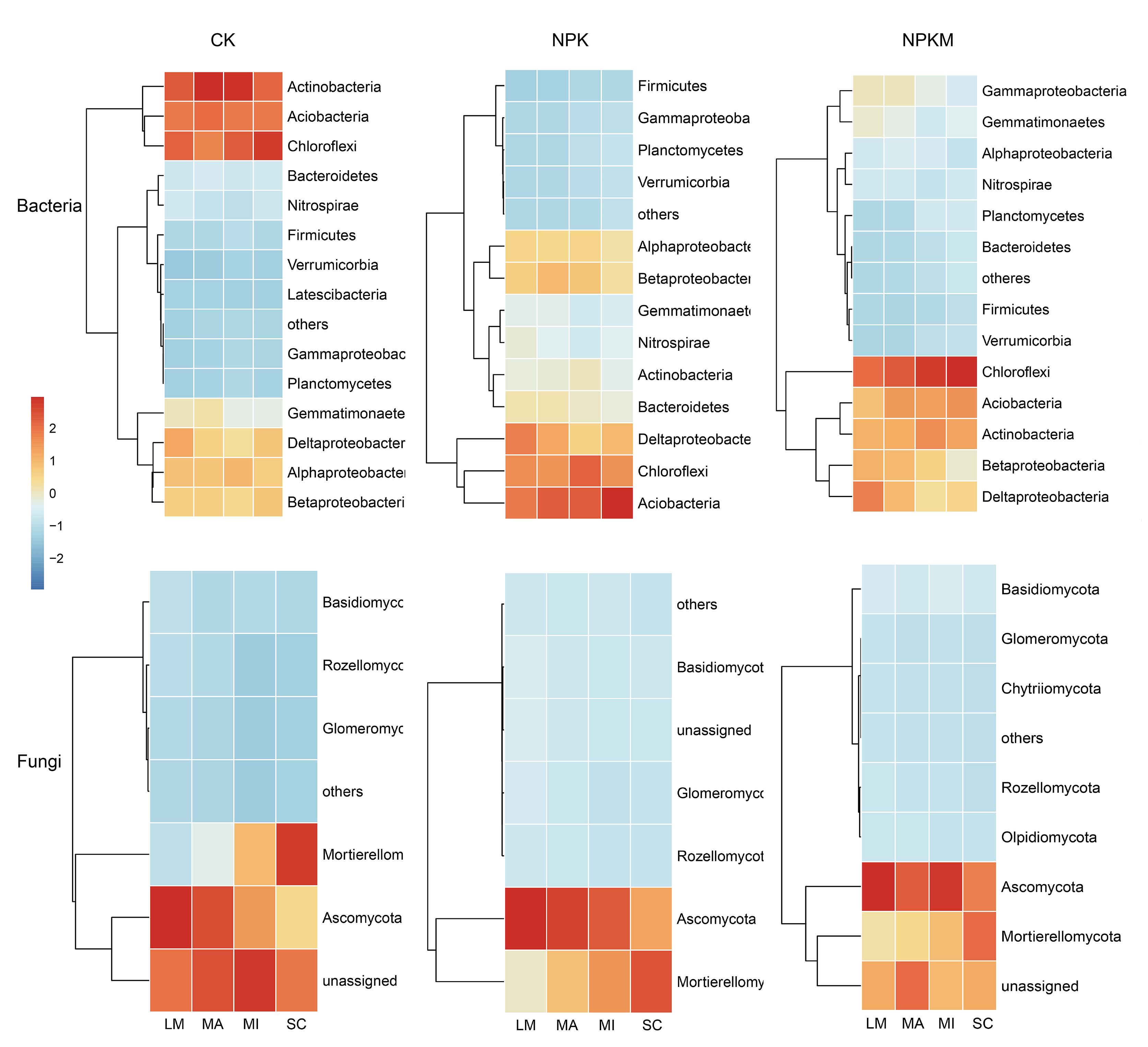

Supplement: Supplementary file 3 [file Presentation_1.zip › Supplementary Figures/FigS10.tif]

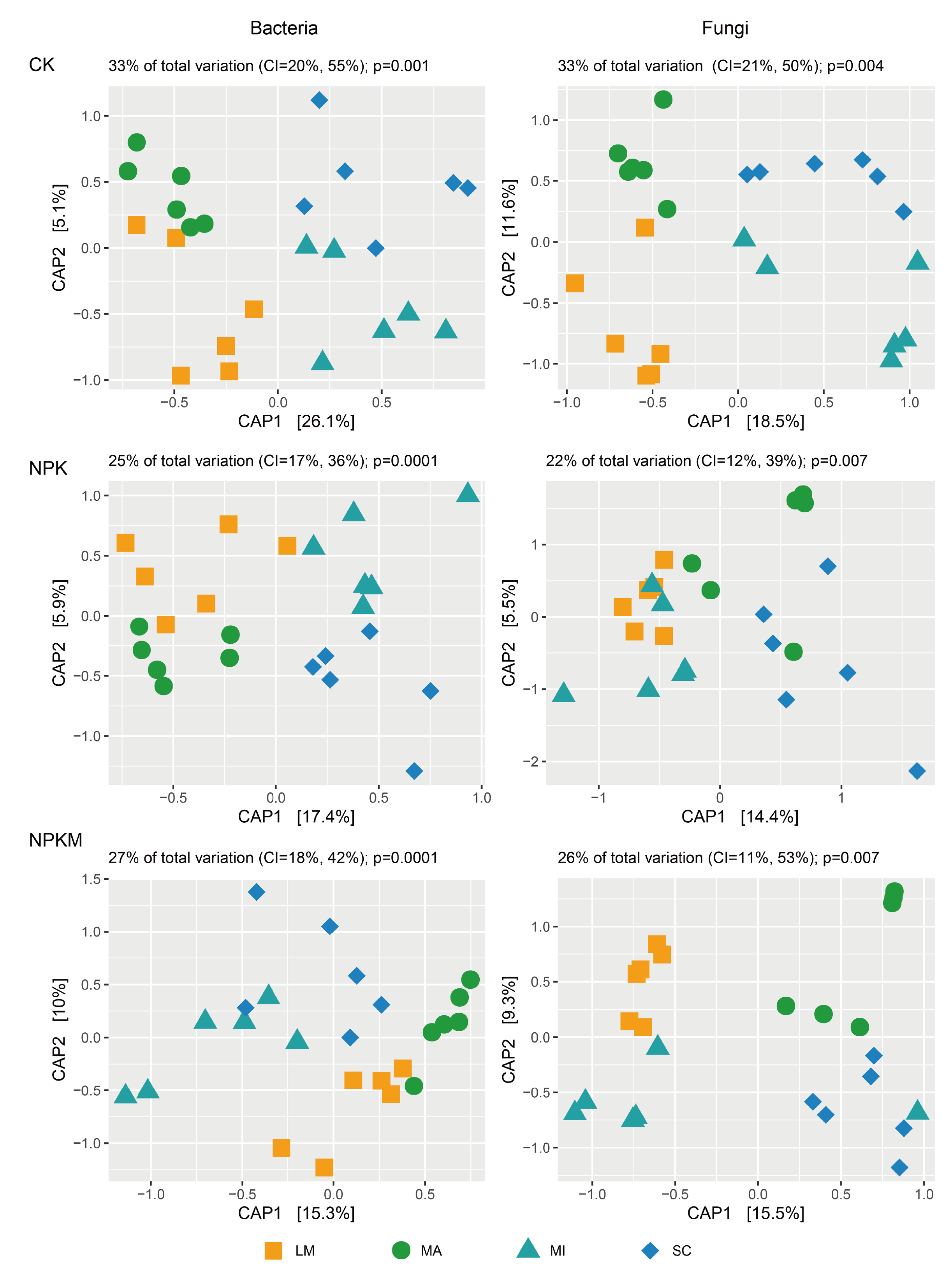

Supplement: Supplementary file 3 [file Presentation_1.zip › Supplementary Figures/FigS2.tif]

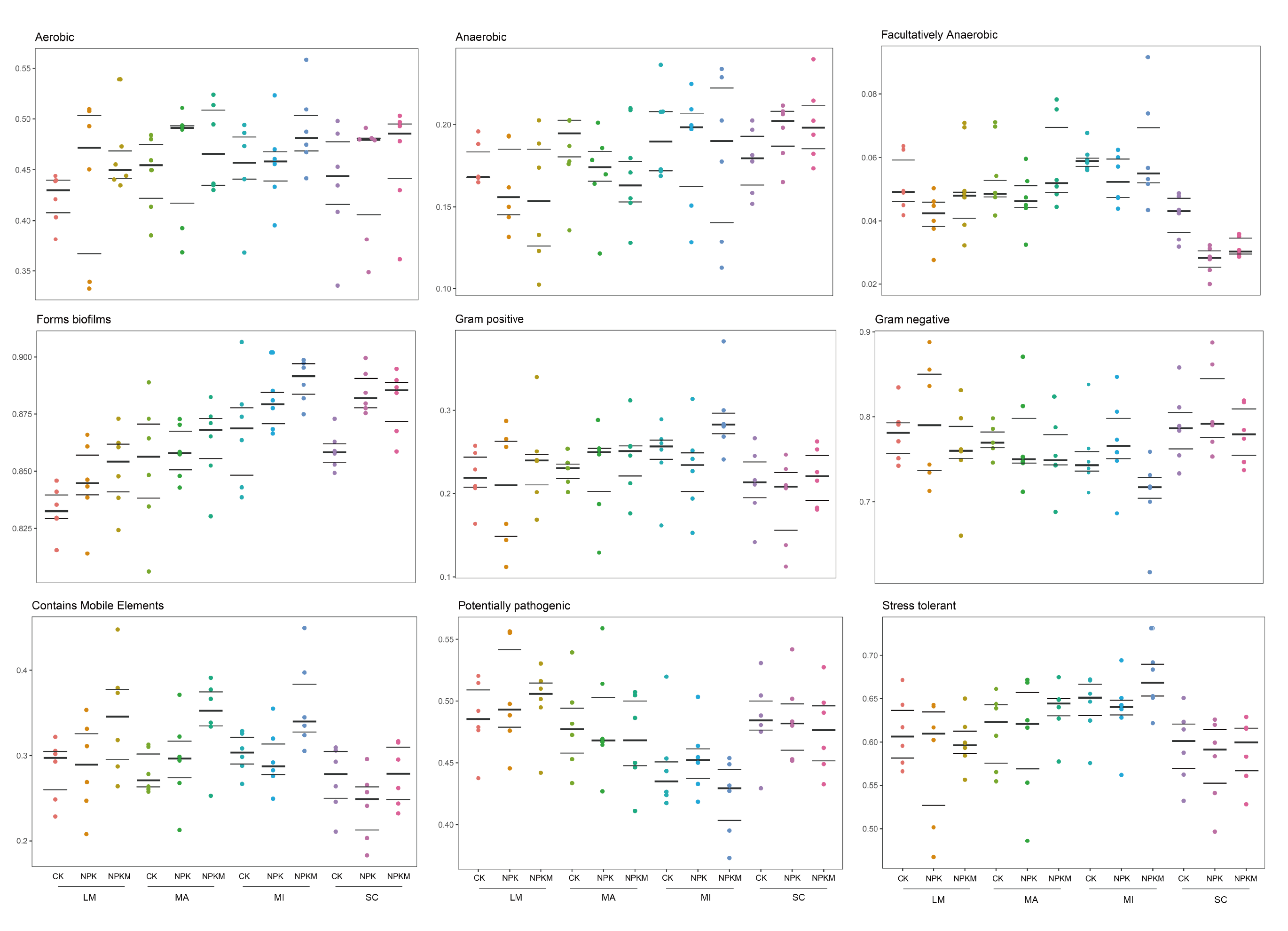

Supplement: Supplementary file 3 [file Presentation_1.zip › Supplementary Figures/FigS3.tif]

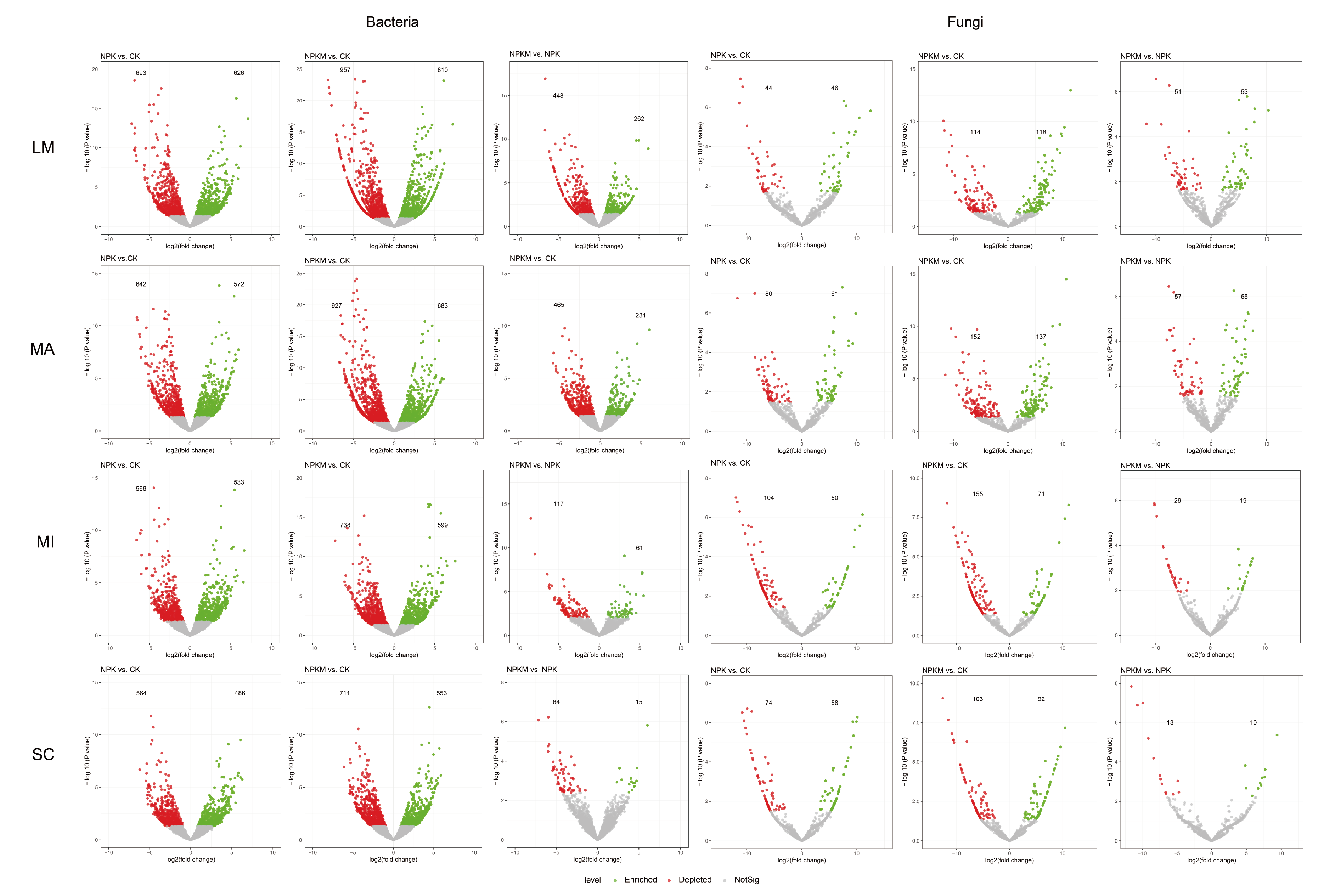

Supplement: Supplementary file 3 [file Presentation_1.zip › Supplementary Figures/FigS4.tif]

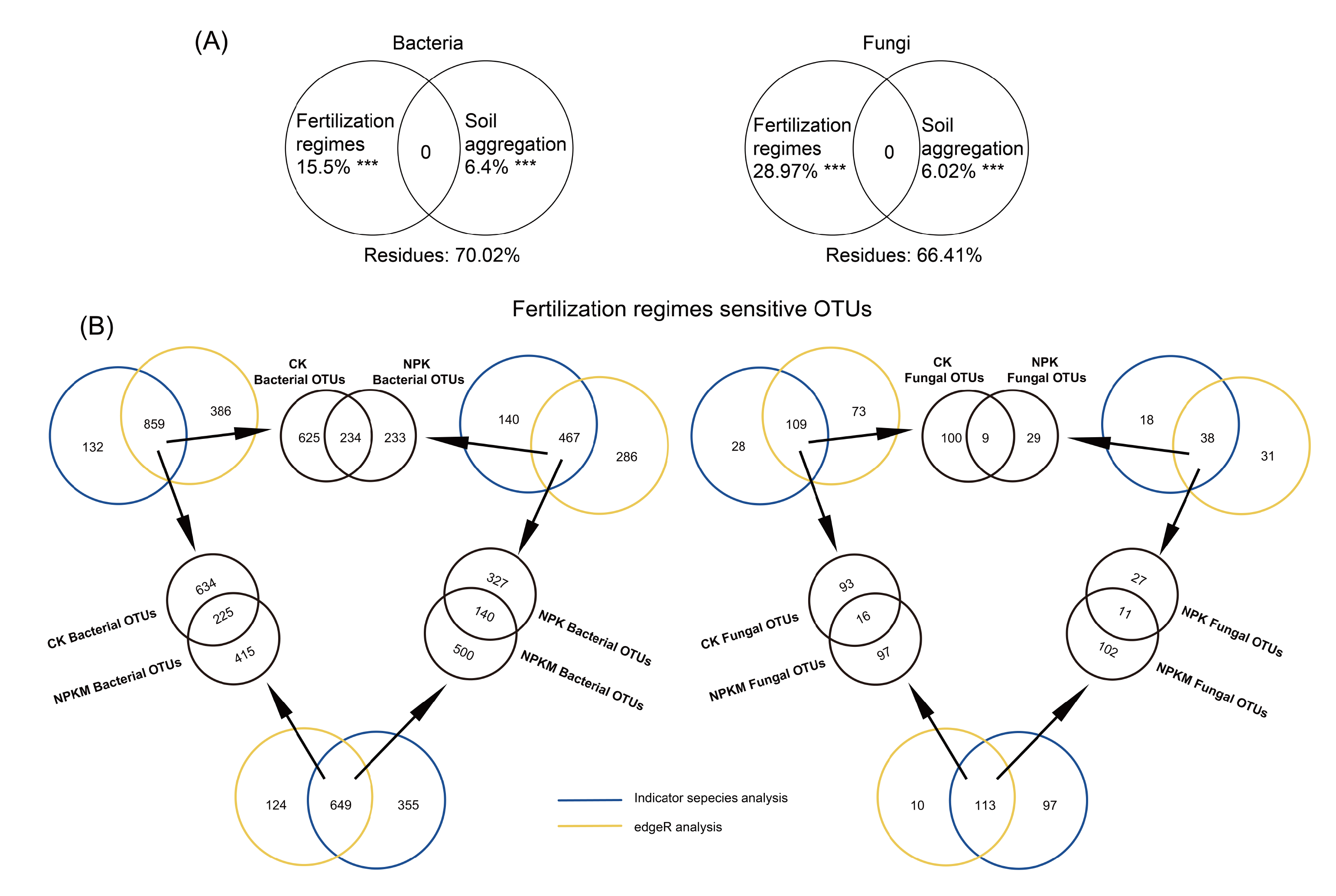

Supplement: Supplementary file 3 [file Presentation_1.zip › Supplementary Figures/FigS5.tif]

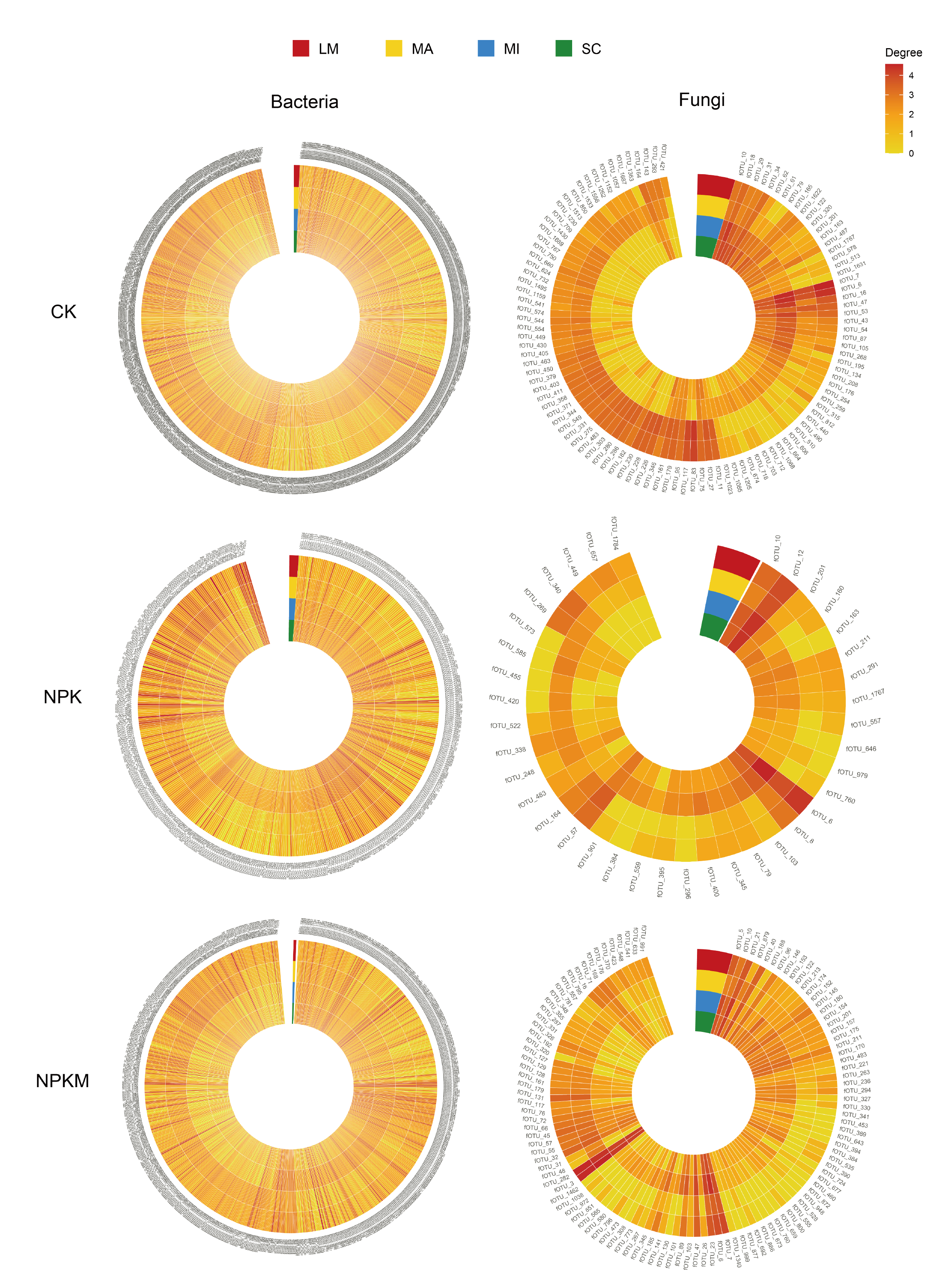

Supplement: Supplementary file 3 [file Presentation_1.zip › Supplementary Figures/FigS6.tif]

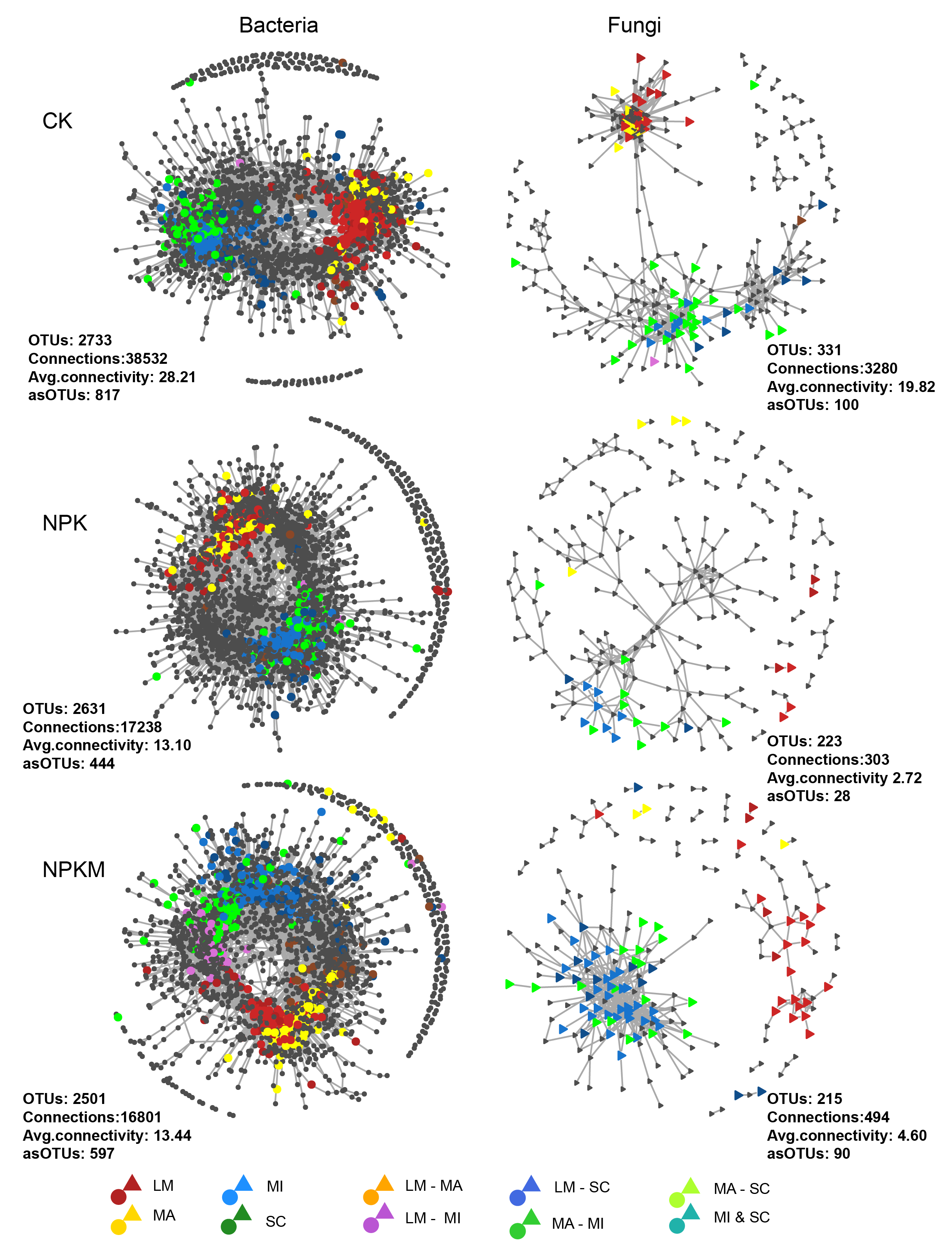

Supplement: Supplementary file 3 [file Presentation_1.zip › Supplementary Figures/FigS7.tif]

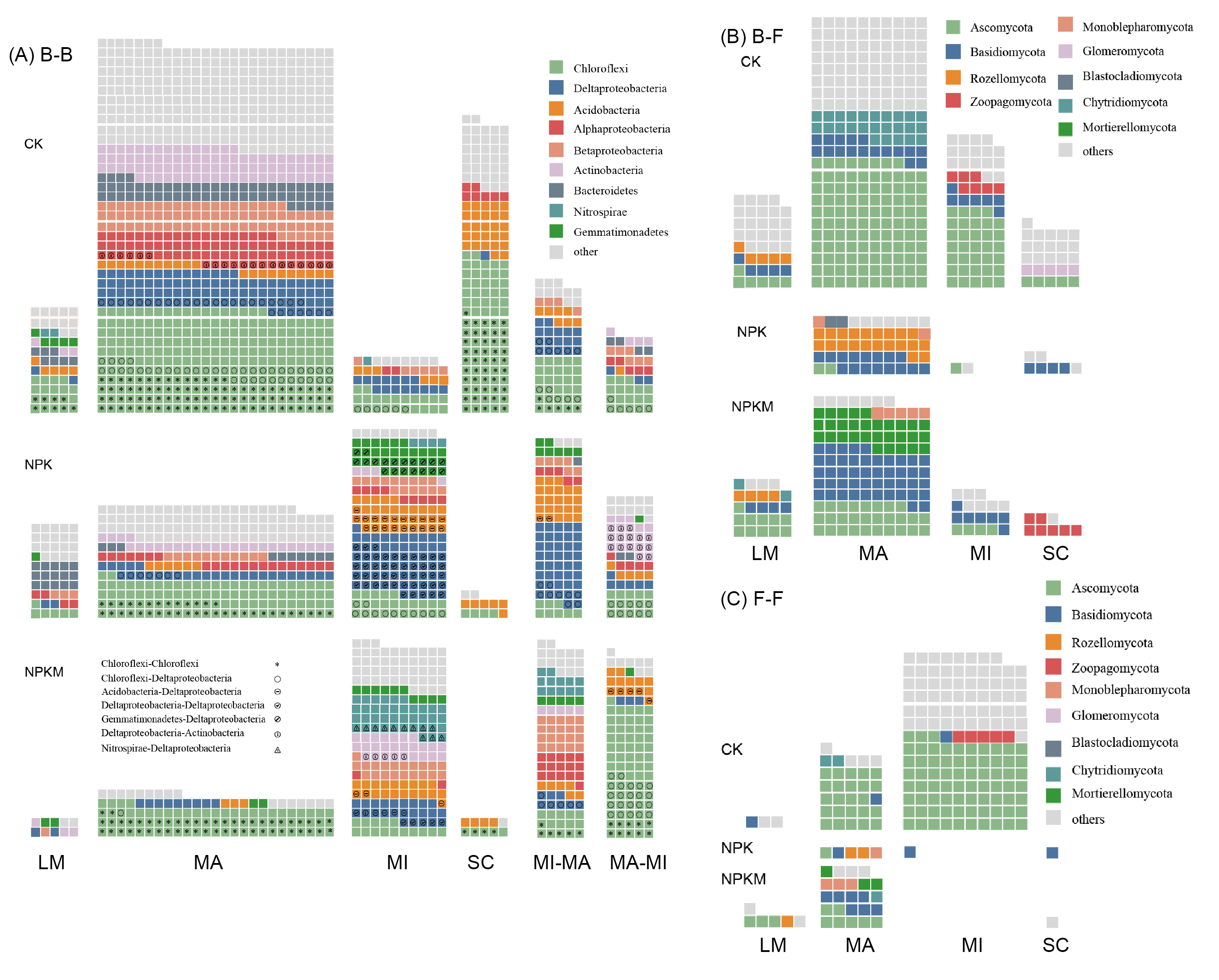

Supplement: Supplementary file 3 [file Presentation_1.zip › Supplementary Figures/FigS8.tif]

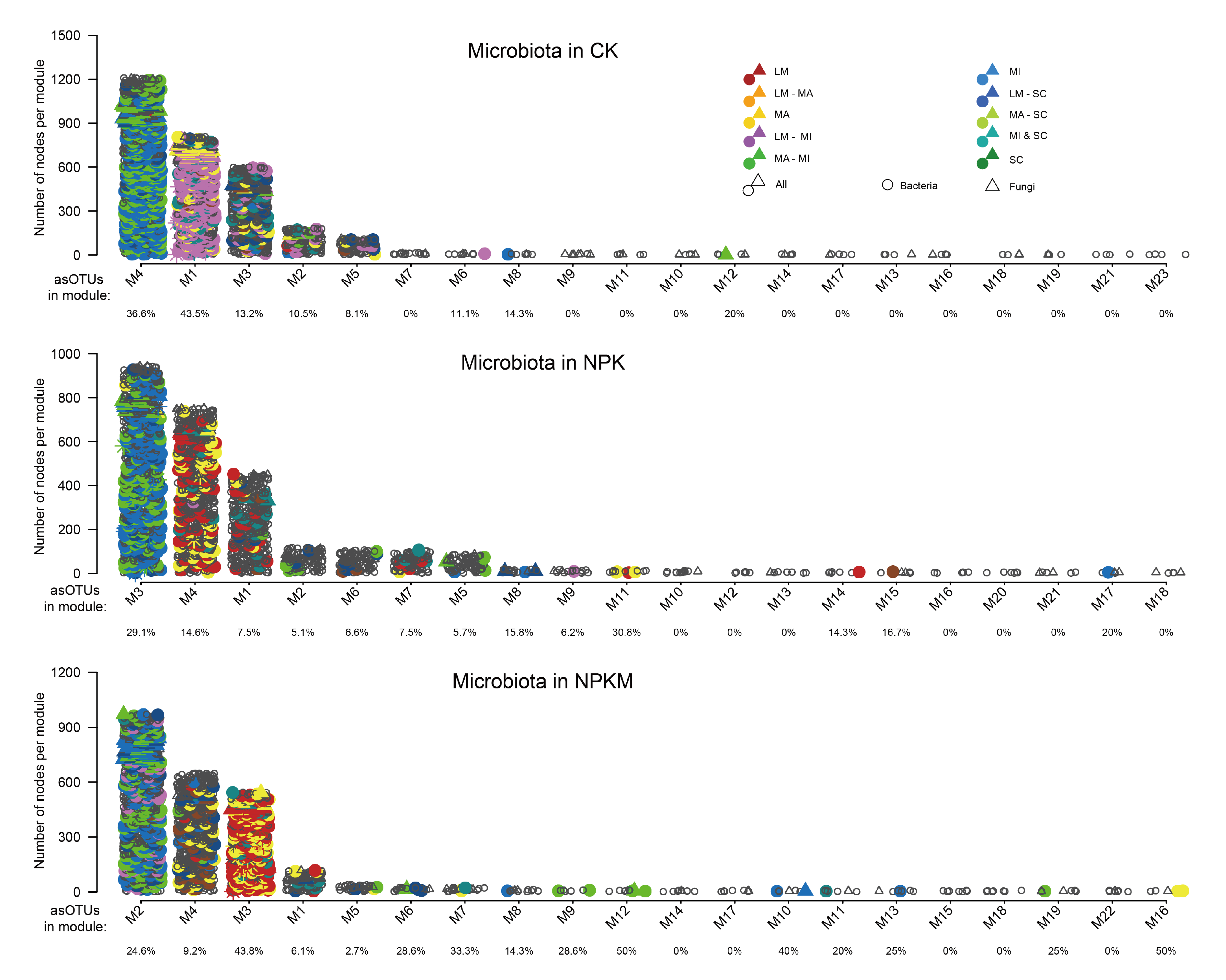

Supplement: Supplementary file 3 [file Presentation_1.zip › Supplementary Figures/FigS9.tif]
